# Pseudoscience is More Dangerous Than Coronavirus Pandemic

**DOI:** 10.34172/aim.2022.104

**Published:** 2022-09-01

**Authors:** Seyyed Muhammad Mahdi Mahdavinoor, Seyyed Hatam Mahdavinoor

**Affiliations:** ^1^Student Research Committee, Faculty of Allied Medical Sciences, Mazandaran University of Medical Sciences, Sari, Iran; ^2^Department of Islamic Theology, Yadegar-e-Imam Khomeini (Rah) Shahre-rey Branch, Islamic Azad University, Tehran, Iran

 Severe acute respiratory syndrome coronavirus 2 (SARS-CoV-2) caused widespread infectious pneumonia in 2019 all over the world. On March 11, 2020, the WHO declared the pneumonia caused by the virus as a pandemic due to the high prevalence of the disease. It quickly spread to all countries of the world and led to uprisings in various countries.^1^

 Governments did their best to combat this emerging disease. To prevent the spread of COVID-19, they enacted strict laws, including the quarantine of cities, compulsion to wear masks, prevention of rallies, and closure of certain businesses.^2^ These measures were costly for governments that spent large sums of money on research to prevent and treat the infection, as well as to help those affected by the pandemic. For example, the US Congress recently passed a $1.9 trillion coronavirus relief package.^3^

 The problems that spread with the outbreak of the Coronavirus pandemic were not limited to the closure of businesses or lack of medicine and medical equipment. In fact, these problems usually occur with the onset of any epidemic or pandemic, and governments devote great efforts to coping with them. The issue that the governments pay little attention to is the proliferation of pseudo-science in the shadow of fear of pandemics. Pseudo-science is a fire under the ashes that ignites with the spread of pandemics and burns the achievements of scientists.

 Science and pseudo-science have been in conflict with each other for a long time. With the enactment of compulsory education law in many countries, pseudo -science has been greatly reduced. For example, most people understand that they should go to a hospital for treatment of epileptic seizures and not to an exorcist.

 With the onset of the COVID-19 pandemic, people in some countries have been protesting against government measures to reduce the prevalence of the disease. People in Iran, France and the United States protested against enforcement of wearing masks, some of them regarded the vaccine as a conspiracy to reduce the number of people, or others believed that microchips were implanted in vaccines to bring people under control. Many also suggested a strange treatment for COVID-19 so that people would turn to them instead of vaccines. In Iran, a mullah, known as Sheikh Abbas Tabrizian who considered himself the father of Islamic medicine advised people to put cotton soaked in violet oil in their anus to treat the COVID-19 infection. Another traditional medicine claimant asserts that drinking camel urine can be a definitive cure for this infection.^4^ In India, some people apply cow dung to protect themselves from the disease or organize festivals in which they drink cow urine.^5^ There are many such cases in the world that exist in almost all countries. This type of treatment, which is not based on evidence and can perhaps be called the product of people’s delusions, has also multiplied among rulers and legislators. For example, former US President Donald Trump suggested that people afflicted with COVID-19 inject disinfectants into their body to beat coronavirus and ‘clean’ their lungs.^6^

 These types of therapies, which usually rely on personal experience to prove their effectiveness, are considered examples of pseudoscience because they try to prove something using a non-scientific method. Such treatments reach their peak during pandemics because many people are willing to seek refuge in anything for fear of their lives even without being aware of relevant dangers. During the COVID-19 pandemic, video clips were posted on social media every day that sought to question evidence-based science and medicine to direct people toward non-evidence-based medicine. One of these clips showed several people whose bodies had become magnetic and absorbed metals after receiving the COVID-19 vaccine. This kind of information infiltrated among the people and caused them to lose their trust in scientific therapies. The risk of pseudo-science outbreaks may not be smaller than that of the COVID-19 pandemic as long-term pseudo-science may indirectly kill more people. Reluctance to receive the vaccine has become one of the biggest problems for the US government. Some US states have offered financial incentives to encourage people to get vaccinated, but many are still unwilling to receive a vaccine. This lack of trust in the vaccine causes unvaccinated people to become infected with COVID-19, and some of them eventually die. There is a similar situation in many other countries.

 The law of compulsory education can be considered as the greatest human endeavor to confront pseudo-science. That effort has been highly useful but not enough. Teaching research methods to graduate students was another factor that converted them to ambassadors of science development.

 It seems that pseudo-science cannot be controlled without spending exorbitant costs on culture and education. To counter pseudo-science, governments must have long-term plans. Here are some suggestions on how to confront pseudo-science:

1. The effect of different variables on the prevalence of science and pseudoscience should be measured. For example, does the use of Facebook during the COVID-19 pandemic play a role in people’s reluctance to get vaccinated? By knowing this and similar information, policymakers will be able to do better planning.
2. Teaching basic and advanced research methods to students of all levels and disciplines, especially medical students. Each of these students will have the potential to confront pseudo-science in the society.
3. Developing strict rules to combat pseudo-science. For example, if someone claims to have developed a drug that can treat people with AIDS, they must prove themselves scientifically and otherwise be punished.
4. In order for a behavior in the society to become a culture, it must be promoted and taught for many years.^7^ Curricula can be developed that simply teach students the difference between science and pseudo-science in schools. Thus, they are less likely to be deceived by pseudo-science in the future, and when confronted with pseudo-science, the students will confront it if it leads to conflict of interest.

These suggestions are just basic ideas that need to be developed. Governments must allocate funds to combat pseudoscience in the long run so that in future pandemics, their people are less likely to be deceived by pseudoscience.
